# Cultural Competence Among Optometry Students at a South African University: Cross-Sectional Survey Study

**DOI:** 10.2196/97974

**Published:** 2026-09-17

**Authors:** Sanele Buthelezi, Gloria Tsholofelo Tamenti

**Affiliations:** 1 Department of Optometry Faculty of Health Sciences University of Johannesburg Johannesburg, Gauteng South Africa

**Keywords:** cultural competence, optometry education, cross-cultural care, health professions education, South Africa, medical education

## Abstract

**Background:**

Cultural competence is an essential dimension of contemporary health care delivery, particularly in diverse, multicultural societies. South Africa, with its rich linguistic and cultural plurality, presents a unique context in which culturally responsive care is critical for equitable health outcomes. Despite growing international attention to cultural competence in health professions education, little research has explored this domain within optometry education in sub-Saharan Africa.

**Objective:**

This study aimed to assess self-reported levels of cultural competence, specifically preparedness, skillfulness, and educational exposure, among third- and fourth-year optometry students at a South African university.

**Methods:**

A descriptive, cross-sectional survey design was used. An adapted version of the Harvard Cross-Cultural Care Survey was distributed via Google Forms to 91 eligible students, yielding 68 (74.7%) responses. Quantitative data were analyzed using descriptive statistics in SPSS (version 29). Qualitative responses were analyzed using conventional content analysis.

**Results:**

Participants reported high preparedness for caring for immigrant patients (47/68, 69.1%) and lesbian, gay, bisexual, transgender, queer or questioning, intersex, and asexual (LGBTIQA+) patients (45/68, 66.2%), but low preparedness for patients with religious beliefs affecting treatment (only 20/68, 29.4% prepared) or health beliefs at odds with Western medicine (21/68, 30.9% prepared). Regarding skills, students felt most capable of assessing illness understanding (58/68, 85.3% skilled) and identifying English literacy (56/68, 82.4% skilled) but reported deficits in counseling patients about traditional and alternative medicine use (45/68, 66.2% lacking adequate skill) and identifying religious beliefs affecting care (42/68, 61.8% lacking adequate skill). Experiential learning, particularly community outreach and the Phelophepa Healthcare Train, was identified as the most impactful preparation for cross-cultural care. Content analysis identified 6 categories related to cross-cultural learning experiences: exposure to diverse patient populations, communication across cultures, developing cultural awareness and empathy, awareness of health inequities, informal and social learning, and gaps in structured training.

**Conclusions:**

The optometry students demonstrated moderate cultural competence, with meaningful strengths in communication and rapport-building, alongside notable gaps in engaging with religious beliefs, traditional medicine, and systemic health inequities. These findings align with international literature and underscore the need for structured, curriculum-embedded cultural competence training. We recommend the integration of formal training modules, reflective practice frameworks, faculty development, and expanded equitable access to diverse clinical placements.

## Introduction

In an increasingly globalized world, cultural competence has emerged as a critical skill in health care. South Africa, as a multicultural, multilingual society with a complex colonial and apartheid history, presents a particularly important context for culturally responsive clinical education. The country’s demographic diversity, combined with significant health disparities along racial and socioeconomic lines, means that health care professionals must be equipped to navigate cultural differences effectively.

Cultural competence in clinical practice is defined as a combination of consistent behaviors, attitudes, and policies within a system or among professionals that enables them to function effectively in diverse cultural settings [1]. It involves recognizing and respecting patients’ diverse values, beliefs, and behaviors, and tailoring care to meet their social, cultural, and linguistic needs [2,3].

Research demonstrates that culturally incompetent care leads to misunderstandings, misdiagnoses, poor patient adherence, and dissatisfaction [4,5]. Conversely, culturally competent health practitioners are more likely to build trust, enhance communication, and foster therapeutic relationships that positively influence health outcomes [6]. Health care systems worldwide are increasingly prioritizing cultural competence training as a core component of professional development [7,8].

However, challenges persist in defining, teaching, and measuring cultural competence effectively across different health care disciplines and contexts. Within optometry specifically, the literature is sparse. Lewandowski et al [9] found that US optometry students were least skilled at recognizing cultural and religious beliefs that could impact clinical care, while Christian et al [10] demonstrated that cultural safety training significantly improved Canadian optometry students’ understanding of power dynamics and cultural bias in patient care. To the authors’ knowledge, no prior study has examined optometry students’ cultural competence in the South African context.

This paper reports on a cross-sectional survey examining self-reported cultural competence preparedness, skills, and educational experiences among optometry students at a South African university. The aim of the study was to assess self-reported levels of cultural competence, specifically preparedness, skillfulness, and educational exposure, among third- and fourth-year optometry students at a South African university and to explore their qualitative accounts of formative cross-cultural learning experiences.

## Methods

### Study Design and Setting

This study adopted a descriptive, cross-sectional design [11] and was conducted at a South African optometry school in Johannesburg. Given South Africa’s complex historical and cultural diversity, the institution offered an appropriate and enriched context for exploring cultural competence among health professions students.

### Participants and Sampling

The study population comprised third- and fourth-year students enrolled in the Bachelor of Optometry program during the 2025 academic year. Convenience sampling was used, as it allowed for accessible engagement with an available student population [12]. Inclusion criteria required participants to be registered in the optometry program, aged 18 years or older, and willing to provide informed consent. Students enrolled in other programs or younger than 18 years were excluded. A total of 91 students were eligible for participation. Data collection took place in March 2025. An anonymous online questionnaire was hosted on Google Forms and distributed via class WhatsApp groups through academic year representatives.

### Instrument

The survey instrument was adapted from the Cross-Cultural Care Survey (CCCS) developed by Green et al [13] at Harvard Medical School. The CCCS is among the few validated tools specifically designed to assess cultural competence within health professions education. The adaptation involved contextualizing items for the South African optometry setting and adding references to locally relevant patient groups (eg, patients distrustful of the South African health care system and the use of traditional and alternative medicine). The instrument was piloted with a small group of students prior to the main data collection, and minor refinements were made based on feedback.

The questionnaire assessed four domains: (1) demographic information, (2) self-perceived preparedness to provide eye care to diverse patient groups (10 items, 5-point Likert scale from “very unprepared” to “very well prepared”), (3) self-assessed skillfulness in cross-cultural care tasks (10 items, 5-point Likert scale from “very unskilled” to “very skilled”), and (4) exposure to cultural competence training and prioritization (13 items using varying response formats: items assessing training exposure and departmental prioritization used a 5-point agreement scale from “strongly disagree” to “strongly agree,” while items assessing direct clinical exposure used dichotomous yes or no response options). A single, standalone, open-ended item, presented separately from the 4 domains, asked students to describe the experiences during optometry school that were most useful in preparing them to care for ethnic minority patients.

### Ethical Considerations

Data collection took place in March 2025, following ethics approval (REC-31373-2024) from the Faculty of Health Sciences Research Ethics Committee. Participation was entirely voluntary, and informed consent was obtained electronically prior to survey completion. Anonymity was maintained throughout; no identifiable information was collected. Participants could withdraw prior to submission without consequence. No compensation was offered or provided to participants for their involvement in the study.

### Data Analysis

Quantitative data were analyzed using SPSS (version 29; IBM Corp). Descriptive statistics (frequencies, means, and SDs) were calculated for all Likert-scale items. For the preparedness construct, response options of “very unprepared,” “somewhat unprepared,” and “somewhat prepared” were grouped as indicating a lack of preparedness, while “well prepared” and “very well prepared” indicated adequate preparedness. For the skillfulness construct, response options of “very unskilled,” “somewhat unskilled,” and “neither skilled nor unskilled” were grouped as indicating a lack of skill, while “somewhat skilled” and “very skilled” indicated adequate skill. This grouping was adopted to create a meaningful binary distinction between students who had attained at least adequate competence and those who had not, consistent with the approach used in the original CCCS [13]. For items within the exposure to cultural competence training and departmental prioritization domain, responses on the 5-point agreement scale were reported across 3 categories: “strongly agree” and “agree” were combined to indicate agreement; “strongly disagree” and “disagree” were combined to indicate disagreement; and “neither agree nor disagree” was retained as a neutral category. Where the neutral category was not reported in the Results text, its omission reflected that responses were either agree or disagree, with the neutral proportion accounting for the remainder, up to 68. Dichotomous yes or no items were reported as frequencies. No item-level missing data were identified in the quantitative dataset, as the online survey platform required a response to each item before submission could proceed. The single open-ended item described above was optional; of the 68 respondents, 52 (76.5%) provided a substantive response. Nonresponses for this item were excluded from the qualitative analysis.

Qualitative responses to the open-ended item (“Describe the experiences during optometry school most useful in preparing you to care for ethnic minority patients”) were analyzed using conventional content analysis [14]. As the item specifically asked about formative learning experiences rather than general clinical experiences of delivering cross-cultural care, the resulting categories describe the educational sources and contexts students identified as contributing to their preparation, rather than their broader experiences of providing care to patients from diverse backgrounds. Responses were read repeatedly to achieve data immersion, after which meaning units were identified and condensed. Codes were derived inductively from the data and subsequently grouped into categories based on shared meaning. No qualitative data analysis software was used; analysis was conducted manually using a structured coding framework developed iteratively by the research team. Analysis was conducted independently by 2 team members and discussed.

To enhance the trustworthiness of the qualitative findings, several strategies were used. Credibility was supported through investigator triangulation, with 2 researchers independently coding the data and reconciling discrepancies through discussion. Member checking was not feasible given the anonymous nature of the survey; however, the final categories were fully discussed by the research team. Transferability was addressed through a thick description of the study context and participant characteristics. Reflexivity was considered throughout the analytic process; as optometry educators embedded in the same institution, the researchers maintained an audit trail and reflective notes to bracket prior assumptions and ensure that categories emerged from the data rather than preconceived frameworks.

## Results

### Participant Demographics

Of 91 eligible students, 68 (74.7%) completed the survey. Most participants were in their fourth year (40/68, 58.8%) and female (49/68, 72.1%). The sample was predominantly Black African (40/68, 58.8%), with White (16/68, 23.5%) and Indian (11/68, 16.2%) students also represented. The majority (46/68, 67.6%) spoke a language other than English as their first language, and most (60/68, 88.2%) were South African nationals. Full demographics are presented in Table 1.

**Table 1:** Demographic characteristics of participants (N=68).

Characteristics			Participants, n (%)
**Level of study**			
	Third year	28 (41.2)	
	Fourth year	40 (58.8)	
**Sex**			
	Male	19 (27.9)	
	Female	49 (72.1)	
**Race or ethnicity**			
	Black	40 (58.8)	
	Indian	11 (16.2)	
	White	16 (23.5)	
	Other	1 (1.5)	
**First language**			
	English	22 (32.4)	
	Other	46 (67.6)	
**Country of birth**			
	South Africa	60 (88.2)	
	Other	8 (11.8)	

### Preparedness for Cross-Cultural Care

Participants reported high preparedness for caring for immigrant patients (47/68, 69.1% well or very well prepared) and patients from the lesbian, gay, bisexual, transgender, queer or questioning, intersex, and asexual (LGBTIQA+) community (45/68, 66.2%). Preparedness was low for patients whose religious beliefs may affect treatment, with 70.6% (48/68) indicating a lack of preparedness (combining “very unprepared,” “somewhat unprepared,” and “somewhat prepared”). Similarly, most students reported low preparedness for caring for patients with health beliefs at odds with Western medicine (47/68, 69.1%), users of traditional and alternative medicine (43/68, 63.2%), patients distrustful of the health care system (40/68, 58.8%), and those with disabilities (39/68, 57.4%). Detailed preparedness distributions across all patient groups are presented in Table 2.

**Table 2:** Optometry students’ self-perceived preparedness to provide eye care to diverse patient groups (N=68).

Patient group	Very unprepared, n (%)	Somewhat unprepared, n (%)	Somewhat prepared, n (%)	Well prepared, n (%)	Very well prepared, n (%)
Immigrant patients	1 (1.5)	1 (1.5)	19 (27.9)	32 (47.1)	15 (22.1)
LGBTIQA+^a^ patients	5 (7.4)	6 (8.8)	12 (17.6)	30 (44.1)	15 (22.1)
Limited English proficiency	2 (2.9)	6 (8.8)	23 (33.8)	27 (39.7)	10 (14.7)
Racial or ethnic minority patients	0 (0)	11 (16.2)	22 (32.4)	20 (29.4)	15 (22.1)
Health care system distrust	5 (7.4)	12 (17.6)	23 (33.8)	23 (33.8)	5 (7.4)
Patients with disabilities	5 (7.4)	13 (19.1)	21 (30.9)	22 (32.4)	7 (10.3)
Complementary and traditional medicine users	6 (8.8)	13 (19.1)	24 (35.3)	20 (29.4)	5 (7.4)
Health beliefs at odds with Western medicine	3 (4.4)	12 (17.6)	32 (47.1)	18 (26.5)	3 (4.4)
Religious beliefs affecting treatment	5 (7.4)	17 (25)	26 (38.2)	15 (22.1)	5 (7.4)
Patients from different cultures	0 (0)	11 (16.2)	22 (32.4)	20 (29.4)	15 (22.1)

^a^LGBTIQA+: lesbian, gay, bisexual, transgender, queer or questioning, intersex, and asexual.

### Cross-Cultural Care Skillfulness

Most students rated themselves as skilled in assessing the patient’s understanding of their illness (58/68, 85.3%), identifying patients’ English literacy (56/68, 82.4%), building rapport with patients from different ethnic backgrounds (49/68, 72.1%), and adapting communication styles to meet patient needs (48/68, 70.1%). However, notable proportions reported a lack of skill (combining “very unskilled,” “somewhat unskilled,” and “neither skilled nor unskilled”) in counseling patients about traditional and alternative medicine use (45/68, 66.2%), identifying religious beliefs affecting clinical care (42/68, 61.8%), and identifying cultural customs relevant to care (41/68, 60.3%). Full distributions are presented in Table 3.

**Table 3:** Optometry students’ self-assessed skills in cross-cultural eye care (N=68).

Skill area	Very unskilled, n (%)	Somewhat unskilled, n (%)	Neither skilled nor unskilled, n (%)	Somewhat skilled, n (%)	Very skilled, n (%)
Assessing patient’s understanding of illness cause	1 (1.5)	3 (4.4)	6 (8.8)	48 (70.6)	10 (14.7)
Identifying patients’ English literacy level	4 (5.9)	2 (2.9)	6 (8.8)	39 (57.4)	17 (25)
Building rapport across ethnic backgrounds	1 (1.5)	2 (2.9)	16 (23.5)	34 (50)	15 (22.1)
Adapting communication style	3 (4.4)	4 (5.9)	13 (19.1)	35 (51.5)	13 (19.1)
Negotiating treatment plans	3 (4.4)	3 (4.4)	10 (14.7)	42 (61.8)	10 (14.7)
Identifying patient health care distrust	3 (4.4)	7 (10.3)	24 (35.3)	25 (36.8)	9 (13.2)
Working with medical interpreters	7 (10.3)	5 (7.4)	17 (25)	22 (32.4)	19 (25)
Identifying religious beliefs affecting care	7 (10.3)	12 (17.6)	23 (33.8)	23 (33.8)	3 (4.4)
Identifying cultural customs affecting care	3 (4.4)	9 (13.2)	29 (42.6)	21 (30.9)	6 (8.8)
Counseling on traditional and alternative medicine use	7 (10.3)	15 (22.1)	23 (33.8)	20 (29.4)	3 (4.4)

### Exposure to Cultural Competence Training and Prioritization

The following results are drawn from selected items within the 13-item exposure and departmental prioritization domain. Preclinical years received mixed evaluations: 44.1% (30/68) agreed they provided relevant preparation, while 41.2% (28/68) disagreed. Clinical years were more positively rated, with 50% (34/68) agreeing they contributed to preparedness. Elective modules received limited endorsement (30/68, 44.1% disagreed with their contribution). Experiences outside or prior to the formal optometry curriculum were rated most positively, with 64.7% (44/68) agreeing that these were the most valuable preparation for cross-cultural care.

Most participants (52/68, 76.5%) reported practical experience caring for diverse patient populations during their training. More than half (37/68, 54.4%) reported receiving cross-cultural training. Most reported positive role models among faculty (44/68, 64.7%) and clinicians on placement (44/68, 64.7%). However, 39.7% (27/68) reported exposure to negative or dismissive attitudes from clinicians, and 41.2% (28/68) from fellow students.

Regarding the integration of cross-cultural care in the optometry curriculum, 47.1% (32/68) agreed that cross-cultural issues were incorporated into teaching and clinical care, while 33.8% (23/68) disagreed. Similarly, 48.5% (33/68) agreed that the department prioritized learning about the care of ethnic minority patients, compared with 27.9% (19/68) who disagreed.

### Content Analysis Findings

Content analysis of open-ended responses identified 6 categories describing students’ most impactful learning experiences for cross-cultural care.

#### Theme 1: Exposure to Culturally Diverse Patient Populations

Direct clinical exposure, particularly through community outreach and the Phelophepa Healthcare Train, was the most frequently cited formative experience. Students valued the intensity and breadth of cross-cultural encounters afforded by these settings, which were perceived as more diverse than the university clinic:

The one that has been the most useful was the 2 weeks spent on the Phelophepa train.

Screening patients outside of the Optometry Clinic helped with exposure to different cultures, beliefs and other things that may affect smooth communication and provision of service between a patient and a practitioner.

This category was the most densely coded across all responses. Students consistently distinguished between routine university clinic encounters and outreach settings, characterizing the latter as more likely to involve patients from rural areas, lower socioeconomic backgrounds, and diverse linguistic and cultural backgrounds. The Phelophepa Healthcare Train, which is a mobile health service operating in underserved South African communities, was singled out by multiple respondents as uniquely impactful, often described as their first sustained engagement with patients from communities very different from their own. These accounts suggested that the volume, variety, and authenticity of patient encounters in outreach settings were the primary drivers of cross-cultural learning, rather than any structured educational intervention embedded within them.

#### Theme 2: Communication Across Cultures

Students described navigating language barriers, using translators, reading nonverbal cues, and adapting explanations as critical cross-cultural skills developed through clinical practice:

Learning to navigate language barriers, interpret non-verbal cues, and provide culturally sensitive explanations has strengthened my ability to deliver patient-centred care.

Communication challenges were a recurring concern across student responses, with language barriers identified as the most immediate obstacle in cross-cultural care. Students reported learning to work with ad hoc interpreters, often family members or bilingual peers, in the absence of formal interpreter services. Several noted developing compensatory strategies such as using simplified language, visual aids, and gestures. Importantly, this learning was described as largely self-directed and incidental rather than the result of structured communication training, suggesting that while students were developing practical skills, they were doing so without guidance in best practice interpreter use or communication frameworks tailored to culturally diverse encounters.

#### Theme 3: Developing Cultural Awareness and Empathy

Clinical encounters cultivated appreciation for individual patient backgrounds and the importance of culturally sensitive rapport-building:

I need to learn to adapt and be somewhat relatable to each person in order to develop rapport and trust necessary for the best possible care and patient outcomes.

Students in this category described a process of perspective-taking that extended beyond surface-level acknowledgment of cultural difference. They articulated a recognition that effective clinical care requires understanding the patient as an individual embedded within cultural, social, and historical contexts. Several responses conveyed a growing appreciation that empathy in optometry practice is not merely interpersonal warmth but a clinically relevant skill that enables more accurate history-taking, better adherence negotiation, and more meaningful patient education. This category suggests that experiential learning produced meaningful attitudinal shifts, even in the absence of formal frameworks for developing cultural awareness.

#### Theme 4: Awareness of Health Inequities

Some students articulated an understanding of structural barriers facing patients from minority groups, linking this to professional responsibility:

Coursework and discussions on health disparities have deepened my awareness of the unique challenges that ethnic minority communities face in accessing eye care.

This was the least frequently represented category, appearing in a minority of responses. When it did appear, however, students demonstrated relatively sophisticated critical awareness, acknowledging factors such as historical distrust of the health care system, geographic and financial barriers to eye care access, and the impact of South Africa’s apartheid legacy on health-seeking behavior. These responses tended to come from students who had engaged with formal coursework addressing social determinants of health or who had encountered particularly stark health inequities during community-based placements. The infrequency of this category across the sample suggests that an equity-oriented lens is not yet widespread in students’ interpretation of their clinical experiences, an observation with direct implications for curriculum design.

#### Theme 5: Informal and Social Learning

Interactions with peers and faculty from diverse backgrounds were widely noted as meaningful contributors to cultural competence, often occurring outside formal teaching:

Socialising with classmates helped me with cultural diversity.

A notable proportion of students attributed meaningful cultural learning not to formal curricular activities but to the informal social milieu of a diverse student cohort. Peer interactions, such as shared meals, group study, and social events, were described as contexts in which students learned about different customs, religious practices, communication styles, and lived experiences. Faculty from diverse backgrounds were also mentioned, with students noting that role modeling by culturally diverse clinicians helped normalize cross-cultural clinical encounters. This finding aligns with broader health professions education literature on the “informal curriculum” and highlights that the diversity of the institutional environment itself is a pedagogical resource, one that operates regardless of formal curricular intent but could be more deliberately leveraged.

#### Theme 6: Gaps in Structured Training

A minority of students expressed concern about the absence of explicit, structured cultural competence training:

There is not really any experience training me to deal with ethnic minorities.

While fewer in number, responses in this category were particularly candid and reflected a sense of unpreparedness that students attributed directly to the absence of formal cultural competence content in the curriculum. These students described feeling that their cultural learning had been largely accidental, contingent on which patients happened to present to the clinic or on personal prior experience, rather than the result of deliberate educational design. Some expressed frustration at the lack of explicit guidance on navigating clinical situations involving traditional healers, religious objections to treatment, or patients with very limited health literacy. Taken alongside the survey data from this study, which showed that only 54.4% (37/68) of students reported receiving any cross-cultural training, this category reinforces the perception of a significant gap between the cultural competence demands of South African clinical practice and the preparation the current curriculum provides.

## Discussion

### Summary of Findings

This study aimed to assess self-reported levels of cultural competence, namely, preparedness, skillfulness, and educational exposure, among third- and fourth-year optometry students at a South African university and to explore their qualitative accounts of formative cross-cultural learning experiences. The principal findings indicate that students demonstrate moderate cultural competence overall, with relative strengths in communication-oriented skills (assessing illness understanding, identifying English literacy, and building rapport) and higher preparedness for immigrant and LGBTIQA+ patients. However, notable deficits were identified in preparedness and skillfulness when encountering patients whose care is influenced by religious beliefs, traditional health practices, disability, or systemic distrust of the health care system. Experiential learning, particularly community outreach and the Phelophepa Healthcare Train, was identified as the primary driver of cross-cultural preparation. Content analysis of open-ended responses identified 6 categories of learning: exposure to diverse patient populations; communication across cultures; developing cultural awareness and empathy; awareness of health inequities; informal and social learning; and gaps in structured training.

### Interpretations, Implications, and Comparisons to Literature

This study, to our knowledge, provides the first evidence of self-reported cultural competence among optometry students in South Africa, revealing a profile consistent with the “precompetence” stage described in Cross et al’s [1] cultural competence continuum. Students demonstrated awareness of cultural diversity and basic communication skills but struggled to engage more deeply when religious, traditional, or systemic health beliefs were involved.

The higher preparedness for immigrant patients and LGBTIQA+ individuals likely reflects the growing social awareness fostered in higher education settings. However, consistent with findings by Lu et al [15] in Taiwan, students may interpret acceptance of diverse patient groups as synonymous with clinical preparedness, rather than possessing advanced, tailored care strategies. The finding that more than 70% of students felt unprepared to manage patients with religious beliefs or health practices at odds with Western medicine echoes Green et al’s [13] findings among US medical students. In South Africa, where traditional healing is prevalent and religious beliefs are central to many communities, this gap carries particular clinical significance. Similarly, preparedness to care for patients with disabilities was limited, aligning with international findings for optometry students [9].

Students’ relatively strong skills in communication-related areas, such as assessing illness understanding, identifying English literacy, and building rapport, were consistent with the South African health professions education literature. Govender et al [16] demonstrated that South African occupational therapy students similarly identified communication as central to patient-centered care. These strengths provide a solid foundation for further development of cultural competence.

The deficit in counseling patients about the use of traditional and alternative medicine (45/68, 66.2% lacked adequate skill) is particularly salient in the South African context, where a substantial proportion of patients consult traditional healers alongside or instead of biomedical practitioners. This gap, also identified among international health professions students [13,17], underscores the need for structured curricular content on respectful negotiation between biomedical and traditional health frameworks.

The finding that students overwhelmingly endorsed experiential learning—particularly the Phelophepa Healthcare Train and community outreach placements—as the most valuable preparation for cross-cultural care is consistent with Matthews and Van Wyk [4], who found that South African medical students considered clinical exposure a primary driver of cultural awareness. It also aligns with Putra et al [18], who found that a supportive clinical learning environment was significantly associated with higher cultural competence among nursing students.

However, findings by Fryer et al [19] caution that clinical exposure alone is insufficient for lasting improvements in cultural competence; structured, guided, and assessed learning experiences are required. The notable minority of students who reported receiving no explicit cross-cultural training highlights this gap.

The presence of negative or dismissive attitudes toward cross-cultural care among both clinicians on placement (reported by 27/68, 39.7% of students) and fellow students (28/68, 41.1%) is a notable concern. Such “hidden curriculum” effects can undermine formal teaching, reinforce stereotyping, and impede the development of professional identity. Buthelezi and Gerber [5] found that ophthalmic dispensing educators at a South African university acknowledged that teaching on cultural competence was often unsystematic and unassessed, a finding that may partly explain students’ perceptions of inadequate training.

The profile described here—moderate preparedness, stronger communication skills than specific cross-cultural competencies, and reliance on experiential over structured learning—closely mirrors that reported in international studies among medical [13,20,21], nursing [22], dental [23], and physiotherapy [19] students. The South African context adds distinct dimensions, particularly the centrality of traditional medicine, high linguistic diversity, systemic distrust of health care among some communities, and the legacy of structural health inequities [24].

### Recommendations

Several curriculum enhancements are recommended. First, formal cultural competence modules should be embedded longitudinally across all 4 years, extending beyond awareness to encompass traditional medicine, religious beliefs, disability, and structural inequities, using evidence-based pedagogical strategies such as role-play, case studies, and reflective exercises [19,25]. Second, equitable access to diverse clinical placements, including outreach programs such as Phelophepa, should be formalized to ensure all students receive cross-cultural exposure rather than relying on incidental access. Third, faculty and clinical supervisor development programs should address the “hidden curriculum” by equipping educators to model culturally sensitive practice and actively counter dismissive attitudes in clinical settings. Fourth, language training and interpreter-use training should be incorporated into the curriculum, recognizing that language barriers are a primary challenge in South African cross-cultural care [16,26]. Finally, structured reflective practice should be facilitated during clinical placements to enable students to process and consolidate cross-cultural learning experiences [27-29].

### Limitations

This study has several limitations. The sample was limited to a single institution (n=68 of 91 eligible), restricting generalizability to other South African optometry programs or international contexts. Convenience sampling via WhatsApp groups introduced potential selection bias, as students who were more engaged or accessible may have been more likely to respond. As with all self-report survey research, social desirability bias may have affected responses, particularly on sensitive issues such as LGBTIQA+ care, traditional medicine, or religious beliefs. The cross-sectional design precludes causal inferences about the relationship between curriculum experiences and cultural competence levels. Finally, the analysis is descriptive; no inferential statistical tests were conducted, and findings should be interpreted as exploratory rather than confirmatory.

### Conclusions

To our knowledge, this study provides the first evidence of self-reported cultural competence among optometry students at a South African university, establishing a baseline against which future curriculum interventions can be evaluated. Moderate overall competence was observed, with notable deficits in areas directly relevant to South African clinical practice, particularly regarding traditional medicine, religious beliefs, and systemic distrust of health care. These findings aligned with international patterns across health professions education and underscored the need for structured, longitudinal, and assessed cultural competence training.

The Phelophepa Healthcare Train and community outreach programs represent valuable and potentially unique experiential learning assets [30,31]. However, their impact must be augmented by formal curricular integration, faculty development, and guided reflective practice to ensure all students develop the cultural competencies required to serve South Africa’s diverse patient populations effectively and equitably.

More broadly, the consistent alignment of these findings with international data suggests that barriers to developing culturally competent practitioners are systemic and warrant attention at the level of health professions education policy and accreditation standards [32]. In a country where cultural and socioeconomic inequities continue to drive eye health disparities, cultivating culturally competent optometrists is a matter of health equity.

## Abbreviations

CCCS: Cross-Cultural Care Survey
LGBTIQA+: lesbian, gay, bisexual, transgender, queer or questioning, intersex, and asexual

## Data Availability

The datasets generated or analyzed during this study are available upon reasonable request.
